# Circular RNAs in human cancer

**DOI:** 10.1186/s12943-017-0598-7

**Published:** 2017-01-31

**Authors:** Yumin Wang, Yongzhen Mo, Zhaojian Gong, Xiang Yang, Mo Yang, Shanshan Zhang, Fang Xiong, Bo Xiang, Ming Zhou, Qianjin Liao, Wenling Zhang, Xiayu Li, Xiaoling Li, Yong Li, Guiyuan Li, Zhaoyang Zeng, Wei Xiong

**Affiliations:** 1 Key Laboratory of Carcinogenesis of Ministry of Health, Xiangya Hospital, Central South University, Changsha, Hunan 410078 China; 2 Key Laboratory of Carcinogenesis and Cancer Invasion of Ministry of Education, Cancer Research Institute, Central South University, Changsha, Hunan 410078 China; 3 Hunan Key Laboratory of Nonresolving Inflammation and Cancer, Disease Genome Research Center, The Third Xiangya Hospital, Central South University, Changsha, Hunan 410013 China; 4 Department of Stomatolog, The Second Xiangya Hospital, Central South University, Changsha, Hunan 410011 China; 5 Hunan Cancer Hospital and The Affiliated Cancer Hospital of Xiangya School of Medicine, Central South University, Changsha, Hunan 410013 China; 6 Department of Cancer Biology, Lerner Research Institute, Cleveland Clinic, Cleveland, OH USA

**Keywords:** Circular RNAs, Cancer, Splicing, Biomarker, Regulation

## Abstract

CircRNAs are a novel type of RNAs. With the newly developed technology of next-generation sequencing (NGS), especially RNA-seq technology, over 30,000 circRNAs have already been found. Owing to their unique structure, they are more stable than linear RNAs. CircRNAs play important roles in the carcinogenesis of cancer. The expression of circRNAs is correlated with patients’ clinical characteristics, and circRNAs play a vital role in many aspects of malignant phenotypes, including cell cycle, apoptosis, vascularization, and invasion; metastasis as a RNA sponge, binding to RBP; or translation. Therefore, it is meaningful to further study the mechanism of interactions between circRNAs and tumors. The role of circRNAs as molecular markers or potential targets will provide promising application perspectives, such as early tumor diagnosis, therapeutic evaluation, prognosis prediction, and even gene therapy for tumors.

## Background

Circular RNAs (circRNAs) are a large group of RNAs that form covalently closed continuous loops. The first circRNA was found in a RNA virus in 1976 [1, 2]. Not long after that, Hsu MT et al. discovered the existence of a circular form of RNA in the cytoplasm of monkey renal CV-1 cells using electron microscopy [3]. In 1996, circRNAs were found to consist of exons from RNA transcripts in human cells [4]. In recent years, an increasing number of circRNAs have been found owing to the development of sequencing technologies, with the RNA-seq technology of next-generation sequencing being the most notable. To date, the total number of known circRNAs has reached up to 30,000.

CircRNAs have long been regarded as an accidental byproduct, resulting from errors during post-transcriptional processing, and recent studies have found important functions of circRNAs in multiple biological processes [5]. CircRNAs can act in a broad range of roles, including as miRNA sponges [6] and regulators of RNA binding protein (RBP) in modulating the expression of protein-encoding genes [7]. Some circRNAs even encode peptides [8]. Thus, circRNA disorders should influence the development of a variety of human diseases, including cancer [9]. Furthermore, the unique structures of circRNAs lead to their insensitivity to ribonucleases. Thus, unlike linear RNAs, circRNA molecules can exist in tissues, serum, and urine. Owing to this characteristic, the discovery of circRNAs as biomarkers for human cancer has been shown to be a tremendous possibility.

### Biogenesis of circRNAs

CircRNAs can be sorted into three types, including exonic circRNAs, intronic circRNAs, and retained-intron circRNAs, which are produced from different cycling mechanisms. During the formation of circRNAs, specific sequences are conserved and sequences from upstream and downstream regions are linked together in a reverse direction, all of which contribute to mature circRNAs by back splicing [10]. Although the efficiency of back splicing is much lower than in linear RNAs [5], there is still a large intracellular abundance of circRNAs due to their stability and relatively long half-life period [11]. The earliest back splicing mechanism is dependent on complementary intron matches [12], which widely exist in the exonic circRNA formation process. CircRNAs structured from this mechanism have special repeated sequences, such as Alu and other short sequences, on their two sides [13, 14]. After transcription into RNA precursors, the exon sequences on the two sides of the cycling region will complementarily match with each other under the promotion of repetitive sequences, such as Alu, to form a circle shape. Following this, the spliceosome binds to the cycled RNA precursor under the effect of U6 and U2. Then, it selectively cuts out the exon in the cycling region by cooperating with the protein complex and the U5 core [15, 16]. At the same time, exon sequences connection are reversely cut, forming mature circRNA.

**Fig. 1:**
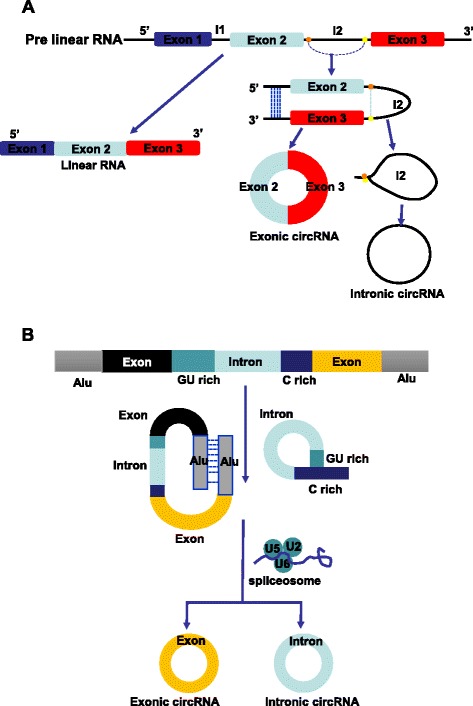
The biogenesis of circRNAs. **a** Alternative splicing of an RNA precursor. A linear RNA precursor can be cis-spliced into linear RNA, including mRNA and lncRNA. Special sequences, such as Alu, in the RNA precursor can combine to form a cycle and lead the spliceosome to produce different types of circRNAs through back splicing. **b** Different circRNAs have different back splicing mechanisms. Exonic circRNAs have cycling sequences, such as the Alu sequence, on the two sides. The two sequences bind to each other complementarily and are then spliced by the spliceosome, which consists of U2 and U6. Intronic circRNAs form a cycle through the combination of upstream introns of the GU rich sequence and the downstream C rich sequence, and they are spliced by spliceosome

The mechanism of intronic circRNA formation is different from that of exonic circRNA. The formation of intronic circRNAs relies on GU-rich sequences near the 5′ splice site and C-rich sequences near the branch point. During the back splicing process, the two segments bind into a circle first. Then exonic sequences and intronic sequences in the binding part are cut out by the spliceosome. The remaining introns are eventually pieced together to form mature circRNA [17, 18]. Recently, Li et al. found another novel type of circRNA consisting of both exons and introns that overexpress themselves according to their complementary sequences [7]; however, the exact splicing mechanism has yet to be determined (Fig. 1a).

Similar to linear RNA, the biogenesis of circRNAs is regulated by various types of proteins, such as the RNA spliceosome and RNA binding protein (RBP). The RNA spliceosome and spicing process are vital research frontiers in biomedicine. The RNA spliceosome is a RNA-based enzyme with a U5 core consisting of 5 snRNAs and a number of proteins. Under the effect the U6 and U2 promoters, many proteins cooperatively process pre-RNAs [16]. Similar to mRNA formation, circRNAs can be spliced from linear RNA precursors by the spliceosome. Furthermore, one linear RNA molecule can be processed into different types of RNAs, including mRNA, circRNA, and lncRNA, through different splicing events. For example, *HIPK3* pre-mRNA can be spliced into *HIPK3* mRNA and its second exon can also form circRNA hsa_circ_0018082 [13]. There is also a competition between inverted splicing of circRNA, mRNA, and lncRNA during splicing [19], which may be a regulation mechanism of circRNAs. Another regulated protein is the Quaking (QKI) protein, a representative molecule. It can bind to specific downstream sites on the linear pre mRNA sequence and promote cycling and inverted splicing of circRNAs [20] (Fig. 1b).

### Databases of circRNAs

With the application of next-generation sequencing and bioinformatics, many circRNAs are being discovered and integrated into the circRNA database [9] (Table 1). The Cirbase and Deepbase 2.0 databases contain over 150,000 circRNA genomes and mature RNA sequences of different species, including human, fruit fly, and nematode [21–23]. The Cir2Traits database contains circRNAs relating to various diseases [24]. Circnet and circRNAbase contain alternatively spliced variants and their expression patterns in different tissues and possible miRNA regulated networks [21, 25]. The CircInteractome database contains corresponding genome sequences, primers for qRT-PCR, and siRNAs of mature circRNAs. It can also be used to predict RBP binding sites [26]. The CIRCpedia and TSCD databases contain RNA-seq data of several human cell lines and samples [27, 28].

**Table 1:** Databases of circRNAs

Database	Developers	Functions of database	Most recent version	Address	Reference
circRNABase	Li J et al.	Constructing a network of predicted interactions between miRNAs circular RNA genes and RBP	December 2013	http://starbase.sysu.edu.cn/mirCircRNA.php	[21]
circBase	Glazar P et al.	Providing merged and unified data sets of circRNAs and the evidence supporting their expression	December 2015	http://www.circbase.org/	[22]
deepBase v2.0	Zheng L et al.	Annotating 14867 human circRNAs.	November 2015	http://deepbase.sysu.edu.cn/	[23]
Circ2Traits	Ghosal S et al.	Constructing a network of predicted interactions between miRNAs and protein coding, long non-coding and circular RNA genes. Disease associated SNPs were mapped on circRNA loci, and Argonaute (Ago) interaction sites on circular RNAs were identified	December 2013	http://gyanxet-beta.com/circdb/	[24]
circNet	Liu Y et al.	Providing novel circRNAs, integrated miRNA-target networks, expression profiles of circRNA isoforms, genomic annotations of circRNA isoforms and sequences of circRNA isoforms	December 2015	http://circnet.mbc.nctu.edu.tw/	[25]
CircInteractome	Dudekula DB et al.	Identifying potential circRNAs that can act as RBP sponges; design junction-spanning primers for specific detection of circRNAs of interest; design siRNAs for circRNA silencing, and Identify potential internal ribosomal entry sites (IRES).	December 2015	https://circinteractome.nia.nih.gov/	[26]
CIRCpedia	Yang L et al.	Containing circRNA back-splicing and alternative splicing from 39 human and mouse samples.	January 2015	http://www.picb.ac.cn/rnomics/circpedia/	[27]
TSCD	He C et al.	Depositing the features of tissue specific circRNAs in the human and mouse genomes.	August 2016	http://gb.whu.edu.cn/TSCD/	[28]

### CircRNAs can be a novel biomarker in cancer

Unlike linear RNA, 3′ and 5′ terminals are not exposed in circRNAs; thus, they are not sensitive to ribonucleases, such as exonuclease or RNase R and have a longer half-life period with a more prominent specificity in histology and pathology [29]. Meanwhile, circRNAs are easier to extract and detect with higher specificity compared with proteins; thus, circRNAs are an ideal molecular marker for cancer. With the application of RNA-seq [12, 30], changes in the expression of circRNAs are found to be prevalent in many cancers, including esophageal cancer [31], glioma [32], and hepatocellular carcinoma [33]. Further experiments and analysis on clinical files confirm that the expression levels of some circRNAs vary according to TNM stage, metastasis, and size the of tumor in esophageal cancer [34], hepatocellular carcinoma [35, 36], and colorectal cancer [37]. Free circRNA molecules are degraded in the extracellular environment, but studies show that circRNAs that are produced intracellularly can be secreted to the exterior of an exosome and be restored after an extended period of time. Li et al. found high expression of circ_0001649 in patients with liver cancer through RNA sequencing and qRT-PCR, and the expression level in the exosome is closely related to clinical characteristics, such as tumor size [33] (Table 2).

**Table 2:** CircRNAs can be novel biomarkers in human cancers

CircRNA	Gene symbol	Alias^a^	Chrome position	Types of cancer	Sample numbers (normal/tumor)	Relative clinical characteristics	Methods	Reference
Tissue								
hsa_circRNA_100855	*C11orf80*	hsa_circ_0023028	chr11:66515849-66590145	Progressive laryngeal cancer	56 (4/52)	T classification; Lymph node metastasis, Primary location, Clinical stage	qRT-PCR	[34]
hsa_circRNA_104912	*DENND1A*	hsa_circ_0088442	chr9:126,438,998-126,641,300	Progressive laryngeal cancer	56 (4/52)	T classification, Differentiation, Lymph node metastasis, Clinical stage	qRT-PCR	[34]
hsa_circ_0001649	*SHPRH*	hsa_circ_0001649	chr6:146209155-146216113	Hepatocellular carcinoma	178 (89/89)	Tumor size; occurrence of tumor embolus	qRT-PCR	[35]
hsa_circ_001569	*ABCC1*	hsa_circ_0000677	chr16:16101672-16162159	Colorectal cancer	24 (12/12)	T classification, Distant metastasis	qRT-PCR	[37]
circ VCAN, circ SMO, circ PLOD2, circ GLIS3, circ EPHB4, circ CLIP2, circ PTN,	*VCAN;SMO;PLOD2;GLIS3;EPHB4;CLIP2;PTN*			Glioma	46 (20/26)	Higher in tumor tissue	RNA seq	[32]
hsa_circ_0005075	*EIF4G3*	hsa_circ_0005075	chr1:21377358-21415706	Hepatocellular carcinoma	66 (33/33)	Tumor size	qRT-PCR	[36]
Exosome								
circ-KLDHC10	*KLDHC10*	hsa_circ_0082333	chr7:129,756,284-129,762,042	Hepatocellular carcinoma	4 cell line	Higher in CRC serum	RNA seq & qRT-PCR	[33]

^a^Alias: the circRNA ID in circBase (http://circbase.mdc-berlin.de)

Because of their unique structure, circRNAs are more stable in tissues, serum, savory, or urine compared with circulating tumor DNAs (ctDNAs) or linear RNAs. There is also a greater possibility to screen for sensitive and specific molecular biomarkers from thousands of circRNAs with regard to the relatively small number of miRNAs. Testing circRNAs by RT-PCR and in-situ hybridization is also more specific and sensitive than detecting proteins by an antigen-antibody reaction. In conclusion, the use of circRNAs outweighs the use of current tumor biomarkers in many fields, and they could be promising molecular biomarkers and new targets for future drugs.

### The function of circRNAs in human cancer

CircRNAs are widely involved in physiological and pathological processes, and studies have found that circRNAs can be involved in biological activities by acting as a miRNA sponge [6], binding RBPs [7], and translating peptides [8, 38]. Of these, the role of circRNAs as a miRNA sponge is the main mechanism of circRNAs in tumor cells. There are many miRNA binding sites on circRNAs. Thus, circRNAs can bind to miRNA as a RNA sponge and can regulate miRNA and downstream gene expression through a ceRNA mechanism and contribute to tumor progression. The following examples present current studies on circRNAs and their functions in human cancer.

#### ciRS-7

ciRS-7, also called CDT1as, is one of the earliest known circRNAs and regulates miRNA in tumor cells [39]. ciRS-7 is spliced from the antisense transcript of the *CDR1* gene. It was first found to be upregulated in malignant tumor cell lines, such as HeLa cells, and further studies found more than seventy miRNA binding sites in ciRS-7 [40]. Later, it was discovered that ciRS-7 can bind to miR7 as a miRNA sponge to downregulate miR-7 expression [41]. miR-671 was found to be another binding site, and the combination of the two molecules mediates the Ago2-mediated cleavage of ciRS-7 to release the absorbed miR-7 [42, 43]. This suggests that miR-671 diminishes the inhibition of miR-7 expression through ciRS-7; improves the miR7 level in tumor cells; contributes to the increase of downstream target oncogenes, such as *EGFR* and *XIAP*; and promotes vascularization, metastasis and reproduction of tumor cells [41, 44].

#### circ-Foxo3

Despite encoding for *Foxo3*, the *Foxo3* gene can encode for two noncoding RNAs, the pseudogene FOXO3P and circ-Foxo3. Yang et al. found that the two RNAs can both act as miRNA sponges for miR-22, miR-136*, miR-138, miR-149*, miR-433, miR-762, miR-3614-5p, and miR-3622b-5p, influencing their function by binding to them. Thus, they are involved in the progression of tumors [45]. Studies have shown that FOXO3P, circ-Foxo3, and Foxo3 mRNA expressed elsewhere can inhibit the growth of a tumor. Further studies have found high levels of circ-Foxo3 in normal cells and that it binds to p21 and *CDK2* to form a circ-Foxo3-p21-CDK2 tripolymer. Thus, in this way, the function of *CDK2* is restricted, leading to a decrease in cell proliferation, and the silencing of circ-Foxp3 will contribute to much faster growth in malignant tumor cells [46].

#### Hsa_circ_001569

Hsa_circ_001569 is a circRNA of 1776 bp that is spliced by linear a RNA precursor from the *ABCC1* gene. The official name of hsa_circ_001569 is hsa_circ_0000677. Liang et al. found that the hsa_circ_001569 had significantly high expression in colorectal cancer tissue and that it correlated positively with the degree of clinical features, such as the TNM stage [37]. This means hsa_circ_001569 may be closely related to colorectal cancer progression. Another study later confirmed this, and hsa_circ_001569 can act as a sponge to bind to miR-145 and inhibit the transcriptional activity of miR-145 without influencing its expression level. Thus, has_circ_001569 leads to the upregulation of target genes of miR-145, including *E2F5, BAG4*, and *FMNL2*, and acts as a positive regulator in cell proliferation and invasion of colorectal cancer.

#### circHIPK3

circHIPK3 is produced by the second exon of the *HIPK3* gene. Huang found higher circRNA levels, including circHIPK3, by RNA sequencing seven types of tumor samples and six types of normal tissues [13]. Silencing circHIPK3 relieves the proliferation speed of tumor cells. Further studies found that circHIPK3 binds to eighteen sites of nine miRNAs. It was found that circHIPK3 inhibits miR-124 activity by working as the sponge of miR-124; thus, it can influence the proliferation of tumor cells through genes, including *iASPP* [47].

#### Fusion-circRNAs

The genomes of tumor cells are unstable; thus, many chromosome translocations exist, especially in lymphoma. Gene fusions may lead to incorrectly spliced mRNAs, during which circRNA (fusion-circRNA,f-circRNA) comes into being. For example, the *PML/RARa* and *MLL* genes fused and produced f-circM9 and f-circPR and knockouts of f-circM9 and f-circPR lead to apoptosis of a large amount of tumor cells as well as increases their sensitivity to drugs, such as arsenic [48]. Together, these results suggest that f-circM9 and f-circPR play important roles in hematological malignancy.

#### cZNF292

Silencing cZNF292 in human endothelium cells will inhibit the tube formation process these cells undergo [49]. cZNF292 also exists in glioma cells [50]. Downregulation of cZNF292 inhibits the proliferation and vascularization of glioma cells and reduces the levels of cellular Cyclin A, CDK2, p-CDK2, β-catenin, p-STAT3 (Tyr705) and p-STAT5 (Tyr694). Thus, the cells are arrested in the G2/M phase due to modifications in the functioning of the Wnt/β-catenin pathway. Meanwhile, it was found that downregulation of cZNF292 contributes to decreased transcription of *E2F1*, *NF-κB*, *Sp1*, *HIF-1*, *AP-1*, *STAT3*, and *STAT5*, thus inhibiting tube formation of tumor cells. However, studies show that cZNF292 does not function as a RNA sponge, and the mechanism requires further study [49].

#### circTCF25

In 467 samples of carcinomas of the urinary bladder, Zhang et al. detected circRNAs with prominent differential expression through microarray analysis. It was confirmed by qRT-PCR that six circRNAs, including circTCF25 (hsa_circ_0041103), were highly expressed in tumor tissues. Another study demonstrated that circTCF25 can upregulate the levels of *CDK6* by working as a RNA sponge for miR-103a-3p and miR-107. Thus, it promotes the proliferation and metastasis of urinary bladder carcinoma [51].

### Prospect

CircRNAs are novel molecules that have been discovered in recent years. They are no longer recognized as products of transcription errors. On the contrary, they have become a popular research subject, after miRNAs and lncRNAs, owing to their important functions [52–56]. The formation of a mature circRNA molecule has at least two mechanisms, which are different from selective splicing in linear RNAs; however, the detailed molecular mechanism requires further studies. There may be a third mechanism involving the formation of retained-intron circRNAs that contain both exons and introns, and this has also yet to be explored. Moreover, different types of proteins and miRNAs have been found to take part in the processing of pre-RNAs to circRNAs. The mechanism of circRNAs formation has yet to be discovered, and it remains an attractive mystery.

A tremendous amount of data indicate that circRNAs regulate other genes at the transcriptional and post-transcriptional levels. Some can even encode proteins directly and are thus involved in physiological and pathological processes, including influencing malignant tumors. Until now, the miRNA sponge has been found to be a possible mechanism for circRNA function. They can specifically bind to miRNAs and regulate gene expression by competing with competing endogenous RNA (ceRNA), including lncRNAs, mRNAs, and pseudogenes. The participation of circRNAs in the ceRNA network renders it more complete and complex. CircRNAs compete with mRNAs from the same preRNAs during the splicing process, and circRNAs were recently found to encode for proteins. This demonstrates their involvement in biological procedures, provides a more complete understanding of the RNA language, and enriches the central dogma [57–59]. Knowledge of circRNAs still remains at a superficial level, and more functions remain to be discovered among over 30,000 molecules, as only a limited number of reports describe their functions. Exploration of their functions will bring humans closer to the essence of life.

CircRNAs are much more stable compared to linear RNAs owing to their unique structure. With regard to biological markers of cancer, circRNAs have demonstrated their wide perspective. Studies have already found differential expression of circRNAs in tissues and blood, and their expression differs from those of miRNA and lncRNA [60–62]. It is possible to build a model for prognosis by combining them with various other biomarkers and thus improve the accuracy and specificity of diagnosis. The clinical applications of circRNAs continues to expand, with their use in samples, such as saliva and urine, and research on circRNAs is indeed a very promising aspect of the study of life sciences.

Previous studies have reported that circRNAs are one type of molecular marker in tumors and that they can influence apoptosis and infiltration. CircRNAs are potential targets for new drugs. For example, the fusion-circRNA found in leukemia is associated with chemotherapy resistance, and knocking out expression of f-circM9 can lead to apoptosis of leukemia cells and decrease its drug resistance to Ara-C [48]. On the other hand, circRNAs perform their functions by mediating miRNAs through a miRNA sponge. This process can also act as a treatment target for malignant tumors. It was also revealed that circRNAs can encode multiple peptides, which can also be used to treat tumors. However, more progress needs to be made between circ-RNA and tumor treatment since the process to effectively and efficiently transform circRNAs into targeted cells is still a major problem that needs to be solved. Although nanoparticles have been applied as transforming vehicles in gene therapy and the Synergistic Photodynamic/Photothermal/Chemotherapy of Cancer drug releasing system has also made some advancement, neither have been utilized in clinical practice. Further studies are still needed to reach a definite resolution.

## Conclusions

In conclusion, current achievements in the study of circRNAs and their functions are still very limited. To our excitement, this mystery will eventually be solved through scientists’ endeavors and applications of new methods and techniques. CircRNAs function in an increasingly significant way in the diagnosis and therapy of cancers and will provide precise strategies for affecting human cancers.

## Abbreviations

ABCC1: ATP binding cassette subfamily C member 1
Ago: Argonaute
BAG2: BCL2 associated athanogene 4
CDK2: Cyclin dependent kinase 2
CDK6: Cyclin dependent kinase 5
CDR1: Cerebellar degeneration related protein 1
circRNAs: Circular RNAs
ctDNAs: Circulating tumor DNAs
E2F1: E2F transcription factor 1
E2F5: E2F transcription factor 5
EGFR: Epidermal growth factor receptor
FMNL2: Formin like 2
FOXO3: Forkhead box O3
HIPK3: Homeodomain interacting protein kinase 3
iASPP: Inhibitor of apoptosis-stimulating protein of p53
miRNAs: microRNAs
NGS: Next generation sequencing
PML: Promyelocytic leukemia
RBP: RNA binding protein
START3: Signal transducer and activator of transcription 3
TCF25: Transcription factor 25
XIAP: X-linked inhibitor of apoptosis
ZNF292: Zinc finger protein 292
